# Chest CT in COVID‐19 patients: A clinical need

**DOI:** 10.1002/jmrs.642

**Published:** 2023-01-02

**Authors:** Brooke Cao, Jonathan Iredell, Peter Middleton, Noel Young

**Affiliations:** ^1^ Westmead Hospital Sydney New South Wales Australia; ^2^ Infectious Diseases & Microbiology Westmead Hospital Sydney New South Wales Australia; ^3^ Respiratory & Sleep Medicine Westmead Hospital Sydney New South Wales Australia; ^4^ Radiology Department Westmead Hospital Sydney New South Wales Australia; ^5^ Faculty of Medicine, Sydney Medical School The University of Sydney Sydney New South Wales Australia

**Keywords:** Chest CT, computed tomography, COVID‐19, pulmonary embolism, thorax

## Abstract

**Introduction:**

The COVID‐19 pandemic caused by the coronavirus SARS‐CoV‐2 has resulted in a global healthcare crisis. The provision of computed tomography (CT) imaging services by radiology departments for COVID‐19 patients poses multiple challenges. Consequently, it is important to explore the clinical need and indications for thoracic CT and whether they subsequently alter patient management.

**Methods:**

We report our experience in this single‐centre retrospective cohort study of all confirmed COVID‐19 cases admitted during the peak of the ‘Delta’ variant wave in Australia, and who underwent a chest CT. Clinical indication and patient management plan pre‐ and post‐CT were ascertained.

**Results:**

A total of 92 out of 1403 patients who were admitted with COVID‐19 underwent a thoracic CT (73 CT pulmonary angiogram (CTPA), 14 CT Chest and five high‐resolution CT (HRCT) studies). 72.8% of studies were to evaluate for pulmonary emboli, 16.2% for assessment of COVID‐19 pneumonia complications, 5.4% for tuberculosis and 6.5% for other indications. 21 (23%) of these studies resulted in a change in management with two patients having a major change in management (thrombolysis, CT‐guided aspiration). Management was altered due to diagnosis of pulmonary embolism (PE), pneumonia, cryptogenic organising pneumonia and other reasons. Of 73 CTPA studies, 11 (15%) patients had evidence of PE.

**Conclusion:**

In our centre, thoracic CT in COVID‐19 patients were predominantly for the evaluation of PE with other indications being for COVID‐19 complications and other cardiopulmonary pathologies. 23% of studies subsequently altered patient management, suggesting there is good clinical need for CT chests for these indications.

## Graphical abstract

Indications of CT chest in COVID‐19 patients during the Delta wave of the COVID‐19 pandemic differ from its usage during pre‐Delta wave. CT chest at our site during the Delta SARS‐CoV‐2 variant wave was predominantly for assessment of other cardiopulmonary pathologies.

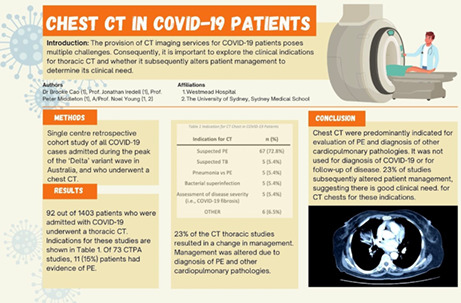

## Introduction

The coronavirus disease 19 (COVID‐19) pandemic caused by the coronavirus SARS‐CoV‐2 has resulted in a global healthcare crisis. The role and utility of thoracic imaging with computed tomography (CT) in terms of the clinical indications and whether it alters patient management is not well established in the current literature.

The provision of CT imaging services by radiology departments for suspected or confirmed COVID‐19 patients poses multiple challenges. These involve the infection control protocols that lengthen the study and the risk of COVID‐19 transmission to radiology healthcare personnel and other patients undergoing imaging.^1^, ^2^ CT studies in COVID‐19 patients have both increased monetary cost such as for personal protective equipment (PPE) to protect healthcare personnel and opportunity cost as less CT studies can be performed in addition to non‐COVID‐19 patients being deferred.^2^

The literature to date (as of December 2021) suggests that CT chest studies should be reserved for evaluating the complications of COVID‐19 pneumonia such as acute respiratory distress syndrome (ARDS), investigating for other cardiopulmonary pathologies such as pulmonary embolism or for patient follow‐up.^1^, ^2^, ^3^, ^4^, ^5^ However, there exists limited literature regarding the clinical indications for CT chest in COVID‐19 patients at a hospital site and whether the scan altered the trajectory of a patient's management. It is imperative to determine this given the resource implications and burden of the provision of CT imaging services. We report our experiences with thoracic CT in COVID‐19 patients.

## Methods

This is a single‐centre‐based retrospective cohort study of all consecutive confirmed COVID‐19 patients during the peak of the ‘Delta’ variant wave pandemic in New South Wales (NSW) (1st July 2021 to 5th October 2021) who underwent CT of the thorax during their hospital admission.

### Participants

The investigator systematically searched the RIS‐PACS (radiology information systems and picture archiving and communication systems) database for confirmed COVID‐19 patients who underwent thoracic CT imaging between 1st July 2021 and 5th October 2021. Inclusion criteria were (1) patient is admitted to hospital, (2) patient underwent CT Chest, CT pulmonary angiogram (CTPA) or high‐resolution CT (HRCT) and (3) confirmed COVID‐19 on polymerase chain reaction (PCR). Exclusion criteria were if the patient had a combined CT chest, abdomen, pelvis scan.

### Data collection

The following data were collected from RIS‐PACS (radiology information systems and picture archiving and communication systems): type of thoracic CT performed which include CT Chest, CTPA or HRCT, clinical indication, positive or negative for pulmonary embolism (PE) if CTPA was performed and whether patient was urgently scanned. Normally, CT scans for COVID‐19 patients were scheduled to be performed after normal hours. We defined a scan urgent if the time of investigation was within 2 h of the CT request time by the ward clinician.

Electronic medical records were also reviewed to record the following data: demographic data including patient sex and age, days post‐COVID‐19 positive on PCR, length of admission at time of scan, if patient was staying in the intensive care unit (ICU) at time of scan and management plan pre‐ and post‐CT chest. A change in management was defined as when a medication was initiated or ceased, the patient was admitted to ICU, or a procedure was performed.

Western Sydney Local Health District (WSLHD) Human Research Ethics Committee approval for this study was obtained.

### CT imaging protocol for COVID‐19 patients

CT scan protocols for COVID‐19 patients have added costs and time involved.^1^, ^2^ At our centre, staff required to transfer patient were a nurse escort, security escort and porter, whilst two radiographers and an imaging nurse were required to be present whilst the procedure was performed. A medical escort was also required for contrast studies if the patient was at risk of deterioration. All staff who entered the scanning room with the patient were required to wear personal protective equipment (PPE) following the correct donning/removing protocols. Essential equipment in the scanning room were covered with plastic sheets and following the scan, a 1‐h terminal deep clean of the equipment and room was performed, and the plastic sheets were disposed.

## Results

### Patient demographic details

There were 1403 patients admitted to our centre with COVID‐19. 92 patients had 98 CT thorax studies in the period between 1st July 2021 to 5th October 2021. For these 92 patients, mean age was 50.35 (SD = 17.41) years, and 52 were male, and 40 were female.

### Clinical information for first CT scan

A total of 18 patients were scanned urgently. There were 73 CTPA, 14 CT Chest and five HRCT studies. 20 patients were in the intensive care unit (ICU) at the time of scan. Demographic details of patients who were scanned urgently versus non‐urgently is shown in Table 1. 72.8% of thoracic CT studies were to evaluate for possible pulmonary emboli, 16.2% for complications of COVID‐19 pneumonia such as COVID‐19 pulmonary fibrosis and bacterial pneumonia and 5.4% for tuberculosis (Table 2).

**Table 1:** Demographic details of patients who underwent an urgent versus non‐urgent scan.

	Urgent CT scan $\left(n=18\right)$	Non‐urgent CT scan $\left(n=74\right)$
Median Age (years)	48 (IQR 36–70)	49.5 (IQR 38–61.3)
Male: Female	2:1	1.2:1
Length of stay prior to CT (days)	1 (IQR 0–6)	2.5 (IQR 1–10)
Days COVID‐19 Positive	8.5 (IQR 4–12)	10.5 (IQR 6–17)
CT Indication		
Pulmonary Embolism $n\left(\%\right)$	13 (72.2%)	53 (71.6%)
Tuberculosis $n\left(\%\right)$	1 (5.6%)	4 (5.4%)
Organising pneumonia vs PE $n\left(\%\right)$	2 (11.1%)	3 (4.1%)
Bacterial superinfection $n\left(\%\right)$	0 (0%)	5 (6.8%)
COVID‐19 Fibrosis $n\left(\%\right)$	1 (5.6%)	4 (5.4%)
OTHER $n\left(\%\right)$	1 (5.6%)	5 (6.8%)
% of CTPA Studies Positive for PE	6.7%	17.2%
Change in Management $n\left(\%\right)$	2 (11.1%)	19 (25.7%)
ICU $n\left(\%\right)$	15 (83.3%)	17 (23%)
Mortality $n\left(\%\right)$	1 (5.6%)	6 (8.1%)

Percentages displayed as a proportion of the patients in the respective patient group. CTPA, CT pulmonary angiogram; PE, pulmonary embolism; ICU, intensive care unit.

**Table 2:** Clinical indications for chest CT 1.

Indication for CT 1	n (%)	Days Post‐COVID‐19 positive on PCR	Admission length (Days)
Suspected PE	67 (72.8%)	9 (IQR 6–15)	1 (IQR 1–6)
Suspected TB	5 (5.4%)	9 (IQR 5–13)	3(IQR 1.5–9)
Pneumonia vs PE	5 (5.4%)	20 (IQR 13–27)	11 (IQR 0–16)
Bacterial superinfection	5 (5.4%)	15 (IQR 2–17.5)	10 (IQR 1–11.5)
Assessment of disease severity (i.e. COVID‐19 fibrosis)	5 (5.4%)	23 (IQR 17.5–28)	17 (IQR 8.5–25)
OTHER	6 (6.5%)	13 (IQR 6.25–16)	5 (IQR 0.75–8.25)

‘Days Post COVID‐19 Positive on PCR’ and ‘Admission Length’ expressed with medians. TB, tuberculosis; PE, pulmonary embolism.

### Clinical implication of chest CT imaging

A total of 21 (23%) of these studies resulted in a change in management with seven of these patients in ICU at the time of the study. Two of 21 of these patients were scanned urgently. Table 3. displays the alterations in patient management after the patients' thoracic CT scan. Two patients had a major change in management (thrombolysis for submassive PE, CT‐guided aspiration for dense pulmonary consolidation) whilst 19 had minor changes. For the patients with minor changes in management, 9/19 had therapeutic anticoagulation for PE, 4/19 were commenced on antibiotics for bacterial pneumonia, 3/19 were commenced on steroids for cryptogenic organising pneumonia and 3/19 for other reasons.

**Table 3:** Changes in patient management post‐thoracic CT.

Change in management	$n\;\left(\%\right)$
Therapeutic anticoagulation for pulmonary embolism	10 (47.6%)
Antibiotics for infective pneumonia	4 (19.0%)
Corticosteroids for cryptogenic organising pneumonia	3 (14.3%)
OTHER (1. CT‐guided aspiration of dense consolidation, 2. tocilizumab initiated for desaturation 2° to COVID, 3. PJP prophylaxis withheld in immunosuppressed patient, 4. Colchicine initiated for suspected pericarditis)	4 (19.0%)

Percentages displayed as a proportion of the 21 patients whose management was altered subsequently to their thoracic CT scan. PJP, pneumocystis jirovecii pneumonia.

Of the 73 CTPA studies, 11 (15%) patients were positive for PE and for 10 of these patients, therapeutic anticoagulation was commenced. Therapeutic anticoagulation was not started for one patient given limited life expectancy (99 years of age). Of these 11 patients, five had central PE, and six had peripheral PE.

### Clinical information for second CT scan

Six patients had a second thoracic CT investigation during their hospital admission whilst COVID‐19 positive, with three of these performed urgently. Table 4 shows these patients' details and the diversity of reasons for a second CT. Four of these patients were in ICU at time of scan. Four (66%) of second thoracic CT scans resulted in a change in management.

**Table 4:** Details about patients who had a second CT.

Patient	Indication of second CT	Days Post‐COVID‐19 positive PCR	Admission length (Days)	Time after first CT (Days)	Change in management
1	Post EKOS™ for bilateral PE to guide further management	17	2	1	EKOS™ catheter stepped down to enoxaparin
2	Suspected PE	9	14	8	Nil
3	Bacterial superinfection	8	8	8	Antibiotics
4	Exclude oesophageal perforation on b/g of pneumomediastinum	1	2	1	Nil
5	PE vs abscess vs pneumothorax	16	16	6	Heparin infusion for positive PE
6	Cryptogenic organising pneumonia	5	22	17	Antibiotics

EKOS™, Ekosonic Endovascular System; PE, pulmonary embolism; b/g, background.

### Mortality

Seven (7.6%) patients died.

## Discussion

The sensitivity and specificity of CT chest in diagnosing COVID‐19 has been found in Boger's 2021 systematic review to be 91.9% (95% CI 0.898–0.937) and 25.1% (95% CI 0.210–0.295), respectively, based on 6 individual studies.^6^ CT chest is not as sensitive as the current standard diagnostic test for COVID‐19 which is RT‐PCR, where a recent meta‐analysis found it to have a sensitivity of 97.2% (95% CI 0.903–0.997) suggesting that using CT alone does not have good clinical utility diagnosing COVID‐19.^6^ However, CT has had an indisputably important role in countries with shortages of RT‐PCR testing kits such as in Wuhan, China during the early months of 2020 COVID‐19 pandemic as it was able to provide a fast and accurate diagnosis.^7^ Currently, the American College of Radiology, Society of Thoracic Radiology and American Society of Emergency Radiology recommend against the use of CT for screening and diagnosis of COVID‐19 pneumonia.^8^, ^9^ The literature to date suggests that CT should be reserved for evaluating severity and complications of COVID‐19 pneumonia such as acute respiratory distress syndrome (ARDS), when an alternative diagnosis such as pulmonary embolism is suspected or for patient follow‐up.^1^, ^2^, ^3^, ^4^, ^5^

Although the role of CT chest in diagnosing COVID‐19 and the manifestations of COVID‐19 on CT have been well studied, the specific indications for requesting a chest CT in COVID‐19 as well as the utility of these findings has been less well established. Current research list scenarios or indications for chest CT in COVID‐19 patients where it would be helpful such as obtaining a baseline image, investigating other cardiopulmonary abnormalities and COVID‐19 progression or follow‐up, discouraging indiscriminate use.^1^, ^4^, ^10^ Regarding the utility of chest CT, there have been studies conducted before the emergence of the ‘Delta’ SARS‐CoV‐2 variant globally, which describe its prognostic value in terms of specific clinical outcomes, that is admission to ICU.^11^, ^12^

Our study quantifies the proportion of different clinical indications for chest CT in COVID‐19 patients, and the utility of these findings defined as a general change in management. The period of study coincides with the ‘Delta’ variant of SARS‐CoV‐2 in our centre, which is thought to be more severe than other variants including ‘Omicron’, now dominant globally.^13^ The provision of CT imaging services by radiology departments for confirmed COVID‐19 patients has multiple challenges. Some of these challenges include the scanning of patients outside of normal operating hours for safety reasons, additional staff involvement and deep cleaning of the scanning room. During the peak of the ‘Delta’ variant COVID‐19 wave period at our centre, CT imaging in confirmed COVID‐19 patients was only requested by infectious diseases, respiratory, intensive care and emergency treating teams or by consulting infectious diseases if the patient was under the care of a different team.

A recent questionnaire in 2021 about CT utilisation in 62 healthcare sites across 24 countries pre‐Delta variant SARS‐CoV‐2 showed that 47% of CT were for follow‐up of findings related to COVID‐19 pneumonia, 39% for diagnosis and 9% for complications.^5^ Our study found that 72.8% of CT chest were for suspected pulmonary embolism, and 16.2% were for complications of COVID‐19 pneumonia including COVID pulmonary fibrosis and bacterial superinfection (Table 2). In comparison with the 2021 questionnaire, no CT chests were for diagnosis of COVID‐19 or follow‐up of patients in our centre. For five patients in our centre, CT chests were requested to assess disease severity as they had prolonged illness associated with long admission times (17 days (IQR 8.5–25)) (Table 2). Thus, the proportion of different clinical indications for requesting CT chests at our centre during the ‘Delta’ SARS‐CoV‐2 wave differed from Homayounieh et al's 2021 questionnaire on CT utilisation.^5^

Our study found that 23% of CT chest studies in COVID‐19 patients lead to a change in management after diagnosing other cardiopulmonary pathologies in most of these cases (Table 3). This suggests that it has good clinical need for the indications listed in Table 2. Interestingly, 25.7% of patients who had a CT scan non‐urgently had a subsequent change in management, compared with only 11.1% in the urgent group (Table 1). This is justified by the fact that 83.3% of patients in the urgent group were in ICU (Table 1) and hence were likely approved for the scan urgently given their clinical condition.

We found that 15% of CTPA studies in COVID‐19 patients who had a median admission length of 1 day (IQR 1–6) were positive for pulmonary embolism. The short median admission length suggests this is consistent with COVID‐19‐associated coagulopathy that has been described in the literature where there is pulmonary micro‐vascular thrombosis and a higher incidence of thrombo‐embolic events leading to pulmonary embolism.^14^, ^15^, ^16^ Studies published before the emergence of the Delta wave SARS‐CoV2 variant have reported an incidence of PE in COVID‐19 patients who had a CTPA investigation as between 22 and 30%.^17^, ^18^, ^19^, ^20^ Certainly, the current literature has shown that there is a high incidence of PE in COVID‐19 patients and our study found that 72.8% of chest CTs were for this indication.

### Limitations

This study is limited by its retrospective nature, small sample size and single‐centre experience.

## Conclusion

In conclusion, 6% of patients in the cohort of COVID‐19 patients admitted to our centre during the Delta variant wave of COVID‐19 in NSW, Australia underwent a CT of the thorax. The indication for CT chest in these patients were primarily for the diagnosis of other pulmonary pathologies. 73% of chest CT scans were for the evaluation of possible pulmonary emboli and 16.2% were for evaluation of COVID‐19 complications. CT chest was not used for diagnosis or follow‐up of COVID‐19 in any of our patients. 23% of thoracic CT studies in our centre subsequently altered the patient management, suggesting that thoracic CT has good clinical utility for similar indications.

## Conflict of Interest

The authors declare no conflict of interest.
